# Accurate Predictions Facilitate Robust Memory Encoding Independently From Stimulus Probability

**DOI:** 10.1162/opmi.a.14

**Published:** 2025-07-26

**Authors:** Jiawen Huang, Eleanor Furness, Yifang Liu, Morell-Jovan Kenmoe, Ronak Elias, Hannah Tongxin Zeng, Christopher Baldassano

**Affiliations:** Department of Psychology, Columbia University, New York, NY, USA

**Keywords:** human memory, schema, prediction, eye tracking, computational modeling

## Abstract

We can use prior knowledge of temporal structure to make predictions about how an event will unfold, and this schematic knowledge has been shown to impact the way that event memories are encoded and later reconstructed. Existing paradigms for studying prediction, however, are largely unable to separate effects of prediction accuracy from effects of stimulus probability: likely outcomes are assumed to be predicted, while unlikely outcomes are assumed to cause prediction errors. Here we use a novel approach in which we can independently manipulate prediction success and stimulus probability, by using real-time eye-tracking when viewing moves in a board game. The moves can be consistent or inconsistent with a participant’s predictions (assessed via fixation patterns) and can be also be likely or unlikely to be played by a strategic player. By decorrelating these two measures, we found that both probability and prediction accuracy boost memory through two separate mechanisms, leading to different eye-movement strategies at retrieval. Accurate prediction improved encoding precision, allowing participants to directly retrieve these moves without the use of schematic knowledge. Probable moves, on the other hand, led to improved memory through a retrieval-time strategy in which schematic knowledge was used to generate candidate moves for recognition. These results shed new light on the specific role of predictions in enhancing event memories, and provide a more realistic paradigm for studying schemas, learning, and decision making.

## INTRODUCTION

Our perception and memories of the world are scaffolded by our prior experiences. We can use schemas, the structured knowledge built from these prior experiences, to make predictions about upcoming events (Clark, 2013; Friston, 2010; Huang et al., 2023). For example, we can predict what characters in a story might do next or whether an athlete is about to score in a game. These predictions have important implications for how we remember events, but the ways in which accurate or inaccurate predictions impact subsequent memory are still controversial. While numerous research studies have shown that prediction *errors* lead to better episodic memory (Antony et al., 2023; Bein et al., 2021; Jang et al., 2019; Quent et al., 2022; Rouhani et al., 2018; Wahlheim et al., 2022), in part through enhanced encoding (Frank & Kafkas, 2021; Neuschatz et al., 2002), previous work found that prediction *accuracy* was associated with better memory (Huang et al., 2023), and learning new information is generally easier when it is *congruent* with prior knowledge (Bein et al., 2015; Brod & Shing, 2019; Quent et al., 2022; van Buuren et al., 2014). In most memory paradigms, however, it is difficult to determine whether making an accurate prediction has a causal effect on memory at all, since the accuracy of predictions (made before the stimulus appears) is almost exactly confounded with the schema-consistency of the stimulus (the probability of the stimulus occurring in the current context). Probable stimuli are more likely to be predicted, and improbable stimuli tend to elicit prediction errors (Quent et al., 2021; Schliephake et al., 2021).

Because these two concepts are so closely related, prior work has largely conflated cognitive processes related to prediction with those related to probability, but in fact these may engage quite different mechanisms occurring at different points in time. Before a stimulus is presented, we can generate predictions about this stimulus based on what we have recently observed. These predictions will often be sparse and incomplete; for realistic events, there is generally an enormous space of possible outcomes and only limited time and cognitive resources available to make predictions. After experiencing the stimulus, we can assess the accuracy of our prediction, and (whether we were right or wrong) we can try to make sense of the outcome by using our schematic knowledge to link it to our prior observations. For example, even a chess Grandmaster will sometimes commit a blunder in a game, especially under time pressure, failing to predict an opponent’s move but immediately recognizing the move as sensible after observing it. An engaging narrative will often include events that we did not predict (Baldassano, 2023), but that in retrospect can in fact be meaningfully integrated into our current event model, such as when a character is revealed to be a villain and we can recognize in hindsight that this is consistent with previously-unexplained events. This dissociation between pre-stimulus predictions and the schema-consistency of the observed stimulus in fact plays a key role in some cognitive theories of humor (Raskin, 1984), which propose that jokes intentionally cause listeners to fail to predict a schema-consistent punchline.

In most lab-based paradigms, however, there are a very small number of possible outcomes, and participants can predict all the outcomes that “make sense” (have high probability). When memory differences are observed between predicted and unpredicted stimuli, it is therefore unclear whether these differences are driven by the match between the stimulus and the pre-stimulus predictions per se (prediction accuracy) or by post-presentation evaluations of the match between the stimulus and the schema (stimulus probability). The current study aimed at investigating the specific impact of accurate prediction on memory, separate from the probability of the stimulus, and the potential mechanisms behind prediction- and probability-related effects. These mechanisms could involve processes during encoding (Bransford & Johnson, 1972) that improve memory precision (Bellana et al., 2021), or reconstruction processes that improve retrieval of specific kinds of information (Anderson & Pichert, 1978). Schematic knowledge can serve as a probabilistic prior, biasing responses to be more schema-consistent (Alba & Hasher, 1983; Bartlett, 1932; Bransford & Johnson, 1972; Cheng et al., 2016; Graesser & Nakamura, 1982; Hemmer & Steyvers, 2009; Huttenlocher et al., 1991; Ramey et al., 2022), or providing retrieval cues that can allow access to weak episodic memories (Qureshi et al., 2014; Watkins & Gardiner, 1979). Recent work in visual scene perception has shown that patterns of visual attention for a repeated image are driven differentially by episodic memory versus schematic knowledge (Ramey et al., 2020), suggesting that eye movements in response to a memory cue could index the degree to which a schema-based retrieval strategy is being used. If prediction accuracy and stimulus probability influence memory differently, different strategies might be used when people encode and recall moves that are probable compared to moves that are predicted.

In this study, we used a paradigm recently developed (Huang et al., 2023), in conjunction with real-time eye-tracking, to manipulate prediction accuracy separately from stimulus probability and to determine when a schema-based strategy was used during retrieval. We used a game called 4-in-a-row, an extension of tic-tac-toe, where two players compete to connect four pieces in a row (in either horizontal, vertical, or diagonal direction) on a 4 × 9 board (van Opheusden et al., 2023). In a previous study, it was found that participants spontaneously engaged in predictive eye movements when trying to encode game sequences shown to them, and that making predictions consistent with the gameplay model was associated with improved subsequent memory. However, move predictions were much more likely to be accurate when the move was probable (according to a model of likely moves during effective gameplay); we therefore could not determine whether generating an accurate prediction had a specific consequence for memory, separate from the probability of the observed move. In the current study, we adjusted the presented moves in real time, controlling both the probability of the move and whether the specific predictions a participant was making for this move was accurate or not (by analyzing eye-movement data in real time). We therefore independently manipulated prediction accuracy and stimulus probability by showing moves that people were predicting vs. not predicting, as well as moves that were probable vs. improbable. We hypothesized that both prediction accuracy and stimulus probability separately contribute to better memory, as reflected in higher accuracy, higher confidence, and faster reaction times.

We additionally developed a method for using eye-movements to detect the use of schematic knowledge at retrieval, allowing us to study the mechanisms by which stimulus probability and prediction accuracy influence recall. We hypothesized that probable moves would be encoded through a lower-precision gist-like representation (Bellana et al., 2021), requiring more reliance on schematic knowledge at retrieval. Current theories, however, provide conflicting hypotheses about the effect of prediction accuracy on recall strategy. Stimuli that do not generate a prediction error might be less salient, leading to a less robust episodic memory trace requiring more schema-based reconstruction at retrieval. Alternatively, generating an accurate representation of a stimulus before it appears could enhance the depth and quality of encoding due to additional encoding time (Naim et al., 2020) or by eliciting a positive emotional response (Lee & Sternthal, 1999).

We conducted two experiments in which participants learned the rules of the 4-in-a-row game and then attempted to remember moves that were generated by combining participants’ in-the-moment eye-movement with probable move locations. Moves separately varied in their probability (their likelihood to occur during a 4-in-a-row game) and the degree to which they were predicted by the participant (based on their eye movements). Across both experiments, we found that prediction accuracy and move probability independently contributed to better memory, but through different mechanisms: accurately-predicted moves were more precisely encoded and could be recalled without relying on the game schema, while probable moves were remembered through a schema-based recall process.

## METHODS

### Participants

For Exp. 1, we recruited participants through the Columbia University RecruitMe platform. They were paid $30 for the completion of the experiment with up to $10 bonus based on performance in both gameplay and memory task. The Experiment took about 2 hours in total (1 hour gameplay + 1 hour memory task). For Exp. 2, we used the Columbia SONA platform to recruit students seeking research participation credit as part of their introductory psychology classes. We reduced the number of games played by the participants to ensure that the whole experiment took less than the departmental limit of 1.5 hours for SONA participants. All participants were over 18 years of age and gave informed consent for the experiment. The experimental protocol was approved by the Institutional Review Board in a R01 University (AAAS0252).

There were 37 participants for the Exp. 1 (29 female, 9 male), all with normal or corrected-to-normal vision. These participants had a mean age of 26.02 (*SD* = 7.49). The racial makeup of the group consisted of 6 who identified as mixed race, 14 as Asian, 4 as Black or African American, and 13 as White, and 1 who declined to answer. The sample size for Exp. 1 was similar to the previous study with this paradigm, and was intended as a proof-of-concept that our paradigm could successfully manipulate prediction accuracy independently from move probability. Exp. 2 was pre-registered as a higher-powered replication with 80–100 participants, which was our estimate of how many participants we could collect from our University subject pool over the course of a full semester. In Exp. 2, a total of 120 participants signed up, and 105 participants completed both the gameplay and memory portions of the experiment. We excluded 9 participants due to failure to follow instructions (*N* = 3) or extreme difficulty with eye-tracking (*N* = 6), often due to participants wearing glasses; note that due to the rules for Columbia’s SONA program, we were not allowed to apply pre-screening criteria for participants to select only participants who did not require glasses to correct their vision. Data from one participant were lost due to technical errors while running the experiment. The final sample consisted of 95 participants: 35 male, 52 female, and 8 whose demographic information was missing. These participants had a mean age of 20.61 (*SD* = 3.82). The racial makeup of this group consisted of 10 who identified as mixed race, 24 as Asian, 7 as Black or African American, 37 as White, and 6 as other.

### Design and Procedure

The study was a two-part experiment. Participants first completed an online task where they learned the rule of the 4-in-a-row game and played 40 games to get familiar with it. The gameplay task was built on Psiturk (Gureckis et al., 2016) and hosted on Heroku (https://www.heroku.com/). In the second part, they came into the lab and complete the eye-tracking portion. Two experiments were conducted, and Exp. 2 design and analyses were pre-registered in AsPredicted (https://aspredicted.org/jv8qv.pdf).

**Figure 1.**
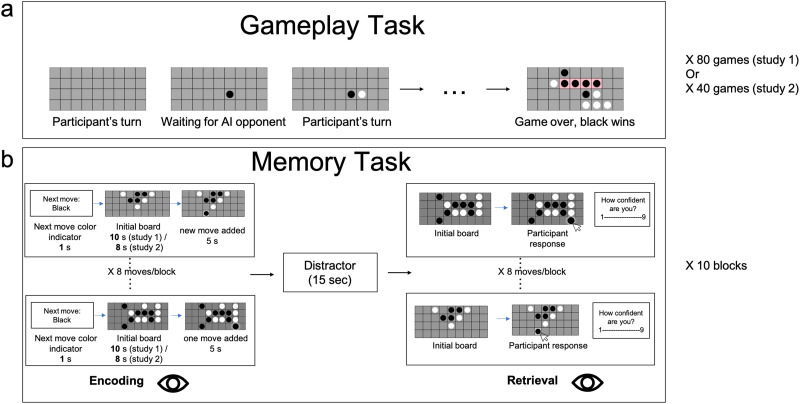
**Experimental design.** **a.** Online four-in-a-row gameplay task. Prior to coming to the lab, participants played 80 (Exp. 1) or 40 games (Exp. 2) against AI opponents. The participant and the AI took turns placing pieces on a 4 × 9 grid, with the goal of connecting four pieces in a row (in any direction) to win the game. **b.** In-lab memory encoding and retrieval task. In each block, participants first went through 8 encoding trials. Each trial indicated which player would make the next move for 1 s, a trial-unique initial game board was shown for 10 (Exp. 1) or 8 (Exp. 2) seconds, and then the next move was added to the board and shown for 5 s. Participants’ task was to remember the single move associated with each board. After seeing all the eight boards and the individual moves associated with each board, participants completed a 15-second distractor task, in which participants judged whether a single digit number was odd or even, participants were shown the initial boards again (in a random order), and recalled the move shown for that board. After they selected the move, the board remained on the screen until participants responded to a prompt underneath the board requiring them to rate their confidence in their response. Participants’ eye movements were tracked throughout both encoding and retrieval.

The procedure of the experiments can be seen in Figure 1. Participants played 80 (Exp. 1) or 40 (Exp. 2) games against an AI opponent taken from (van Opheusden et al., 2023). The gameplay was completed 1–7 days before the day of their scheduled in-person session. The game is an extension of tic-tac-toe, in which two players take turns to add a move on a 4 × 9 board. The first player to connect four pieces of theirs in a row (either horizontal, vertical, or diagonal) on the board wins. Participants were told how to win the game, and then played games against an adjustable AI opponent. When the participant won, the AI became stronger, and vice versa. Participants and AIs took turns moving first. They were told the current level of the opponent at every game. They were told to achieve a target level that represents a strong player and (in Exp. 1 only) that their bonus depended on the level they reached.

For the eye-tracking portion of the experiment, participants came in to the lab and signed the consent form. They were given the overall instruction that: “To give you an overview of the task - in this experiment you will remember and recall moves appearing on 4-in-a-row boards. This task is about 60 minutes and can be quite difficult, so you should try to use your knowledge about the game to help you. The instructions for how to complete the tasks will be on the screen.” Participants were seated 100 centimeters in front of a monitor and placed their heads in a chin rest 45 centimeters away from the eye-tracker. They were instructed to remain as still as possible while the eye-tracker was running and were told that they could take breaks during the experiment in between blocks. Before beginning the experiment and when the participants returned from their breaks, the eye-tracker calibration and subsequent validation were done using a nine-point grid. We recorded right eye movement using EyeLink 1,000 plus at 1,000 Hz recording frequency. Light levels remained constant for the duration of the 60 min memory portion of the experiments. The stimuli were displayed on a 24-inch LED monitor, with a resolution of 1920 by 1080 pixels and a refresh rate of 60 Hz. The outputted EDF files were converted to asc files and parsed with PyGaze (Dalmaijer et al., 2014). The experiment was programed with PsychoPy.

Each of the ten blocks in the memory task consisted of eight trials. For each trial, participants first saw text for one second indicating which color player would be playing this move. This is followed by an initial board for 10 (Exp. 1) or 8 (Exp. 2) seconds, taken from the middle of a game between two AI players as in (Huang et al., 2023), and then the move was added to the board and shown for 5 s. The number of pieces on the board ranged from 4 to 31, with a median of 13 and an average of 13.52 pieces (*SD* = 5.97). Participants were instructed to watch and remember single moves placed on different boards. In Exp. 2 they were additionally explicitly instructed to try to guess the location of the move and move their eyes to that location before it appeared. This instruction was designed to ensure that participants’ fixations on empty squares indeed reflected their predictions. We additionally changed how moves were generated in Exp. 2 to encourage participants to make more predictions with their knowledge of the game (more details in Real Time Generation of the Move section in Methods). After all eight moves were shown, there was a 15-second distractor task in which random one-digit numbers flashed at the center of the screen every 1 s and participants were instructed to a button every time an even number appeared. The distractor task was designed to be relatively short because the task of remembering eight moves is highly challenging even at short retention intervals. Participants were then shown the eight initial boards again, one by one, in random order, and recalled the move that was placed on each board by clicking on the corresponding location. After each recall, they also rated their confidence about the move on a scale of 1 to 9 (with 9 being most confident) by pressing the keyboard.

A persistent technical issue with the eye-tracker sometimes caused the experiment to freeze at random points in the middle of the experiment during both experiments. In case this happened, we continued the experiment from the next block and did not include data from that block in that participant. As a result, 9 participants in Exp. 1 and 13 participants in Exp. 2 had one or two out of the ten blocks missing from their data. The data in the intact blocks of these participants were used for the analysis.

#### Fixation Smoothing and Gameplay Model.

We used the same approach for processing fixations and generating probabilities from the gameplay model in our previous study (Huang et al., 2023).

Fixation maps were created for each 10- or 8-second period before a move was shown. To handle uncertainty in assigning gaze to squares, we performed a soft assignment to board locations based on distance. For a fixation at position *x*_*F*_ with duration *t*_*F*_ the square with center coordinate *x*_*i*_ was assigned a fixation weight of

$$t_{F}\cdot \frac{e^{-{\left\|x_{F}-x_{i}\right\|}_{2}/25}}{\sum_{j}e^{-{\left\|x_{F}-x_{j}\right\|}_{2}/25}}$$

Here, distance is in the unit of pixels. The length of the square is 180 pixels so the smoothing on the scale of 25 pixels is only relevant for fixations close to square boundaries. The weights for all fixations during the 10- or 8-second window were summed to obtain a final map of fixation weights for all board squares.

The gameplay model is a linear myopic model based on features manually selected for gameplay, trained on games played by strong AI with PyTorch (Paszke et al., 2019). The model outputs a probability distribution of likely next moves. A full description and validation of this model can be found in our previous study (Huang et al., 2023).

#### Real Time Generation of the Move.

**Figure 2.**
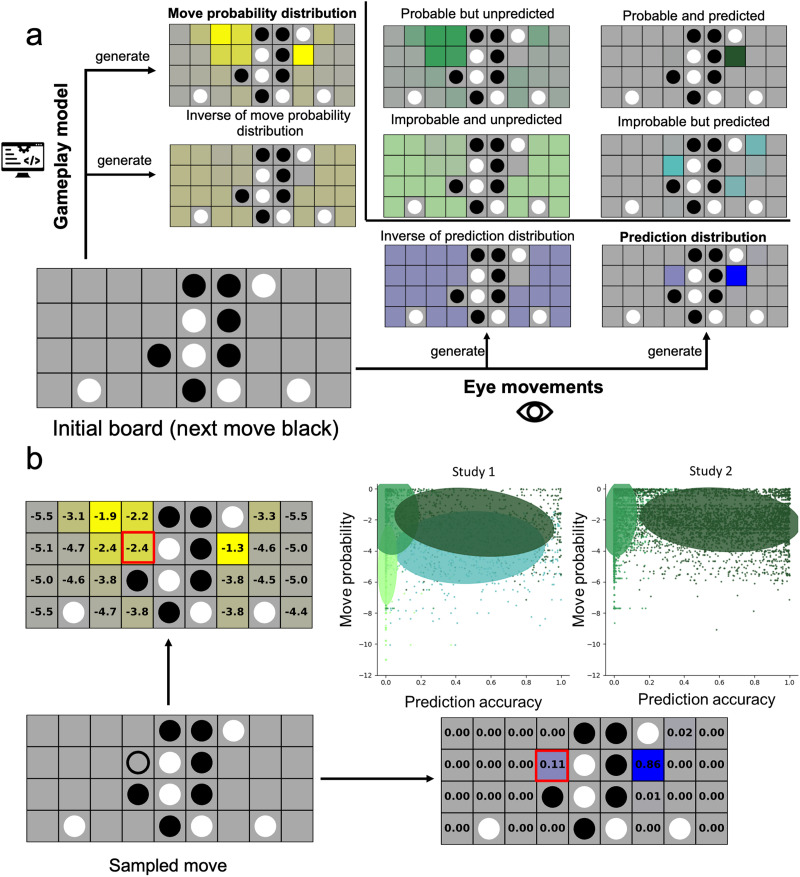
**Real-time generation of the moves**. **a:** After the initial board was shown, two distributions were produced: one based on gameplay model that computes the probability of the move to be played by a strong player (yellow), and one based on the participant’s predictive fixations during the initial board period (blue). Darker colors indicate higher values in the squares. The move probability and prediction distributions or their inverses were then combined to generate four possible distributions from which the presented move was drawn: improbable and unpredicted (used in Exp. 1 only), improbable but predicted (Exp. 1 only), probable but unpredicted (both experiments), and probable and predicted (both experiments). A move was sampled from one of the distributions. **b:** Distribution of move probability and prediction accuracy in each of the four or two conditions in Exp. 1 and 2. After a move was sampled, it was evaluated with the move probability distribution and the prediction distribution. This generates a move probability and prediction accuracy for each move (in this example, move probability is −2.4 and prediction accuracy is 0.11). Each point in the scatterplot is a move, and the color of the points correspond to the distribution from which the move was drawn. The ellipses represent the 3σ confidence ellipses for each condition, with colors corresponding to the conditions in (a), showing where most of the points in each condition are located. The correlation between prediction accuracy and move probability is close to zero.

To generate a distribution of a participant’s predictions (**prediction distribution**, Figure 2a, bottom right) for a board, we obtained a fixation heatmap during the initial board period (10 or 8 s depending on experiment). Then, the fixations on the empty squares were extracted and normalized such that the fixations sum to one. Similarly, we generated a **move probability distribution** (Figure 2a, top left) using the gameplay model.

To produce an unpredicted / improbable condition, one or both of these distributions could be inverted, such that the probability of a move *P*(*m*) became:

$$\frac{\mathrm{Max}\left(P\left(m\right)\right)-P\left(m\right)}{\sum_{i=0}^{n}\left(\mathrm{Max}\left(P\left(i\right)\right)-P\left(i\right)\right)}$$

where *n* is the number of empty squares on the board. This makes the most probable/predicted moves the least likely to occur, while the rest of the moves (originally with near-zero likelihood to occur) became roughly equally likely.

The final distribution for the next move was calculated by multiplying the two distributions together and normalized such that the distributions sum to one.

$$P\left(m\right)=\frac{P_{\text{probability}}\left(m\right)\times P_{\text{prediction}}\left(m\right)}{\sum_{i=0}^{n}\left(P_{\text{probability}}\left(i\right)\times P_{\text{prediction}}\left(i\right)\right)}$$

Distributions corresponding to four conditions were generated: probable and predicted, probable but unpredicted, improbable but predicted, and improbable and unpredicted. The move shown to the participants was sampled according to its probability in one of these combined distributions. In Exp. 1, each block contained two moves drawn from each of the four distributions. One potential downside of this design is that this results in many improbable moves, which may discourage participants from using their general knowledge about the game. In Exp. 2, we instead sampled moves only from the two probable conditions, with half of the moves predicted and half unpredicted. All of the distribution calculations were completed at the end of the initial board presentation period of each individual trial, allowing the presented move to be selected based on the computed distribution.

#### Key Measures.

For our main analyses, we ignored the binary condition labels under which moves were generated and instead measured prediction accuracy and move probability values for each specific move (Figure 2b). Move probability is the log probability according to the gameplay model of the move that was shown. Prediction accuracy is the percent of fixations at empty squares that were focused on the move that was shown. Both move probability and prediction accuracy were z-scored within each Experiment, across all participants and all moves.

We also derived a model-based measure of schema-based eye movements at retrieval (Figure 4a). The fixations during the retrieval period were extracted and converted to heatmaps as described above. For each move, fixations on empty squares were extracted and normalized to sum to 1. We then ran a linear regression with the extracted fixation heatmap as the outcome variable, and two regressors: one consisting of the probability distribution over possible next moves according to the gameplay model, and another that had a value of 0 everywhere except at the square corresponding to the correct move, where its value was 1. The resulting coefficients for these predictors were *w*_*moveProb*_ and *w*_*correctMove*_.

#### Statistical Models.

We first z-scored all the variables (other than RT and confidence, which are more intuitive in their raw form), such that the betas reported in the paper reflect the effect size in terms of how many SD changes in outcome variable is related to changes in one SD of the predictor variable. All statistical models were fit in R with the lme4 Package (Bates et al., 2015). We started with the most complex model with random subject effects for all regressors. In cases where the models did not converge, we simplified the models to reduce the number of random effect terms, until only random intercepts remained in the model.

For the mediation analysis, the significance of the mediation was computed with the package *mediation* (Tingley et al., 2014) that uses a bootstrapping procedure. Standardized indirect effects were computed for each of the 10,000 bootstrapped samples, and the 95% CI was computed by determining the indirect effects at the 2.5th and 97.5th percentiles.

#### Pre-Registration and Data Sharing.

The experimental design and potential analysis were pre-registered in AsPredicted (https://aspredicted.org/jv8qv.pdf) before we started data collection for Exp. 2. The results below present a simplified version of the pre-registered analysis plan; see the supplementary material for full results from the planned analyses in the pre-registration. We also conducted additional exploratory analyses looking at how retrieval eye movements are related to the probability of the selected move, to better understand what this measure means in terms of retrieval strategies. All the data and the code can be found online.

## RESULTS

### Manipulation Check

In our previous study, it was found that experienced participants spent more time fixating on empty squares where a move was likely to appear in typical play (based on a game model); since the actual moves shown were drawn from this same distribution, this led to a substantial correlation between prediction accuracy and move probability (*r* = .228, *p* < .001; Huang et al., 2023). We also found this relationship in the current experiments; computing the mean correlation between fixation on empty squares of the initial board and the probability of the moves for each subject, a one-sample *t*-test across subjects showed that these correlations were significantly above 0.(Exp. 1: Mean correlation = 0.273, *t*(36) = 25.92, *p* < 0.001, Cohen’s *d* = 4.20; Exp. 2: Mean correlation = 0.354, *t*(94) = 43.91, *p* < 0.001, Cohen’s *d* = 4.51), meaning people tend to make predictions on probable moves. Despite the correlation, our real-time procedure for selecting moves allowed us to separately control the extent to which moves were likely and were predicted. Figure 2b shows the distribution of move probability and prediction accuracy for all the moves in the experiment, demonstrating that our conditions successfully sampled from different portions of this space and decorrelated prediction accuracy from move probability in both Exp. 1 (*r* = .074) and Exp. 2 (*r* = −.057). While these correlations are still significantly different from 0 due to the very large number of moves in the experiments (*p* < .001), they are much smaller than in our previous study (and have different signs in each experiment), allowing us to more rigorously estimate the independent impact of move probability and prediction accuracy on memory. Because moves are sampled probabilistically, they vary within and across our four conditions in terms of both their probability to occur and the degree to which the participant predicted them on this trial. For the rest of the paper, we focus on move probability and prediction accuracy for each move as continuous-valued predictors rather than treating each condition as a discrete category.

### Move Probability and Prediction Accuracy Both Improve Subsequent Memory

Although the task is difficult, requiring participants to remember 8 moves associated with 8 unique boards, overall performance was reasonably high with a mean accuracy of 0.49 (*SD* = 0.19) in Exp. 1, and 0.55 (*SD* = 0.15) in Exp. 2. This is much higher than the level of performance that would be achieved if participants were guessing randomly (Mean accuracy 4.95%). We also simulated the mean accuracy if participants used the gameplay model to guess the next move, instead of their episodic memory, which gave us accuracy still lower than the accuracy of the participants (Exp. 1: 25%; Exp. 2: 31%), suggesting that they indeed used episodic memory to complete the task. The mean confidence (out of 9) is 5.65 (*SD* = 1.24) in Exp. 1 and 6.11 (*SD* = 1.19) in Exp. 2. The mean reaction time is 8.3 s (*SD* = 3.33) in Exp. 1 and 7.34 (*SD* = 2.35) in Exp. 2.

**Figure 3.**
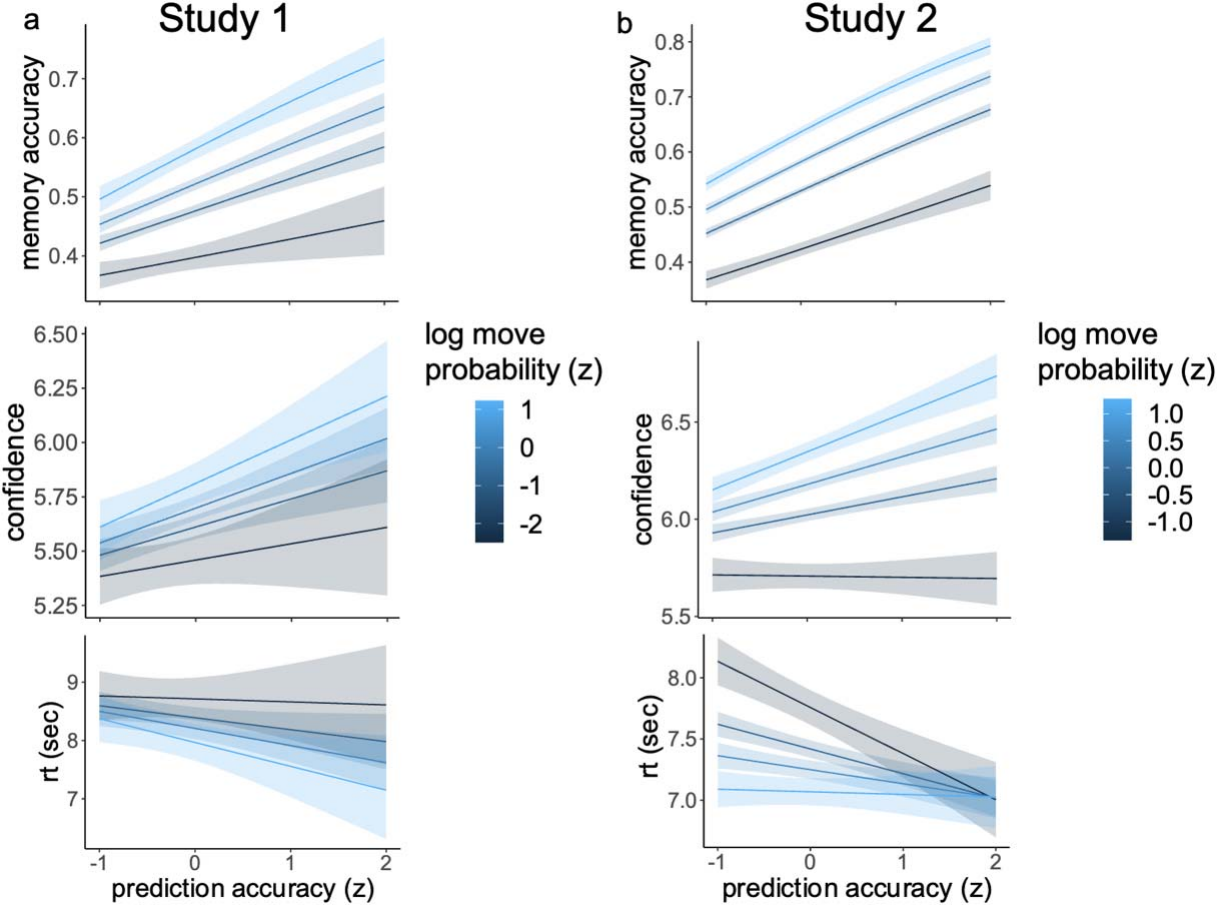
**Impact of move probability and trial-wise predictions on recall performance.** Regression analyses were used to model memory accuracy (top), memory confidence (middle), and reaction time (bottom) as a function of both move probability and prediction accuracy in Exp. 1 **(a)** and Exp. 2 **(b)**. In both experiments, memory was significantly more accurate and confident for more likely moves and when predictions were accurate. These two factors also improved reaction times, though this effect was significant only in Exp. 2.

We first conducted a mixed-effects logistic regression to predict memory accuracy from move probability, prediction accuracy, and their interactions with random subject-specific slopes for move probability and prediction accuracy (Figure 3, top). In both experiments, memory was better for moves that were probable (Exp. 1: beta = 0.233, *z* = 4.64, *p* < .001; Exp. 2: beta = 0.379, *z* = 10.07, *p* < .001) and for moves that were correctly predicted (Exp. 1: beta = 0.289, *z* = 5.19, *p* < .001; Exp. 2: beta = 0.334, *z* = 11.47, *p* < .001). The interactions were not significant in either Exp. (*p* > .30).

We next looked at the effects of both measures and their interactions on memory confidence and reaction time (Figure 3, middle and bottom). Due to convergence issues, these analyses were performed with linear mixed-effects models predicting confidence and reaction times from move probability and prediction accuracy, with random intercepts only. In both experiments, participants were more confident about their answers when the move probability was higher (Exp. 1: beta = 0.09, *t*(2879.5) = 2.08, *p* = .04; Exp. 2: beta = 0.258, *t*(7164.5) = 7.90, *p* < .001) and if the prediction accuracy was higher (Exp. 1: beta = 0.159, *t*(2881.2) = 2.90, *p* = .004; Exp. 2: beta = 0.111, *t*(7168.0) = 4.04, *p* < .001). Both move probability and prediction accuracy led to faster reaction times in Exp. 2 (move probability: beta = −0.268, *t*(7167.8) = −3.60, *p* < .001; prediction accuracy: beta = −0.170, *t*(7172.1) = −2.71, *p* = .007). Similar results were found in Exp. 1 but the effects were not statistically significant (move probability: beta = −0.200, *t*(2881.0) = −1.35, *p* = .18; prediction accuracy: beta = −0.362, *t*(2883.7) = −1.93, *p* = .054). None of the interactions between move probability and prediction accuracy were significant (*p* > .31). These findings provide strong evidence that both move probability and prediction accuracy separately and causally contribute to stronger memories that are recalled faster and with more confidence.

Since prediction errors could benefit memory through enhanced encoding when remembering something schema-inconsistent (Quent et al., 2022), the relationship between prediction accuracy and memory accuracy could be non-linear. To test this hypothesis in our data, we conducted a mixed effect logistic regression predicting memory accuracy from move probability and prediction accuracy with linear and quadratic terms for both effects, and a random subject intercept. While the linear effects for both measures remained robust in both experiments (all *p* < .001), quadratic effects were largely absent. The square of prediction accuracy showed no effect on memory (*p* = .38 in Exp. 1 and *p* = .92 in Exp. 2). Move probability squared did show a significant effect in Exp. 2 (beta = .05, *z* = 1.99, *p* = .047) and trended towards significance in Exp. 1 (beta = .03, *z* = 1.71, *p* = .087), but this effect was weak compared to the linear effect; critically, memory still improved monotonically with move probability (with an attenuated slope for low-probability moves).

### Eye Movements Reveal Multiple Distinct Retrieval Strategies

**Figure 4.**
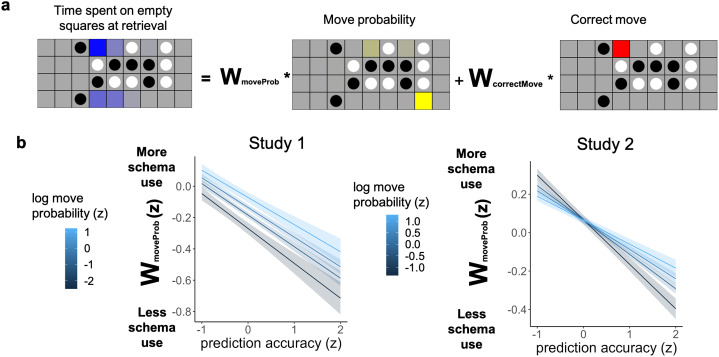
**Modeling and predicting eye movements at retrieval.** **a:** We predicted the distribution of fixations on empty squares when boards were presented at retrieval, using two regressors: the likelihood of each move according to our gameplay model, and the location of the correct move that was shown during encoding. The coefficient of the move probability regressor measures the extent to which schema-based eye movements were present at retrieval. **b:** In both experiments, when we evoked a prediction error during encoding, we observed more schema-driven eye movements at retrieval. In Exp. 1, moves that were more probable also showed more schematic eye movements; in Exp. 2, this relationship only held for moves that were accurately predicted.

We next sought to understand the strategies that participants are using at retrieval by modeling their eye movements. It’s possible that participants could use their schematic knowledge about likely moves for a particular recall board, either by generating plausible moves for the given board then attempting to recognize which move was previously seen (Watkins & Gardiner, 1979) or by simply biasing their guesses toward probable moves. Alternatively, if participants formed a precise episodic memory for a move on a board, they could directly retrieve this memory when the board is shown, without having to make use of schematic knowledge. To use eye movements to detect when participants were using a schema-based strategy at retrieval, we ran a linear regression to predict retrieval fixations on empty squares as a combination of two regressors: (1) the move probability of each empty square (including the correct next move) and (2) the correct next move (with a value of 0 in all other squares) (Figure 4a). The coefficient of the move probability regressor (*w*_*moveProb*_) represents how often a participant was fixating on likely moves during retrieval for this trial, indicating schema use. Note that, because the correct move is included as a nuisance regressor, simply making fixations on the correct move will not increase the value of *w*_*moveProb*_.

Since we would expect participants to use their schema at retrieval more when they are more uncertain, we validated that *w*_*moveProb*_ reflects schema use by checking whether it was associated with worse memory. We conducted three mixed effect regressions predicting memory, reaction time, and confidence from *w*_*moveProb*_. For memory accuracy, we conducted a mixed effect logistic regression, with random slope of *w*_*moveProb*_. For reaction time, we conducted a linear mixed effect regression, with subject random intercept. For confidence, we conducted a linear mixed effect regression, with subject random slope of *w*_*moveProb*_. Overall, we confirmed that larger *w*_*moveProb*_ was associated with worse memory: more schematic eye movements predicted lower memory accuracy (Exp. 1: beta = −0.67, *z* = −7.14, *p* < .001; Exp. 2: beta = −1.18, *z* = −17.98, *p* < .001), higher reaction times both when considering all moves (Exp. 1: beta = 0.47, *t*(2896.2) = 2.76, *p* = .006; Exp. 2: beta = 0.36, *t*(7180.0) = 5.86, *p* < .001) or when restricting to correct moves only (Exp. 1: beta = 0.76, *t*(1419.9) = 4.87, *p* < .001; Exp. 2: beta = 0.59, *t*(3945.0) = 6.09, *p* < .001), and lower confidence for all moves (Exp. 1: beta = −0.40, t(26.0) = −5.11, *p* < .001; Exp. 2: beta = −0.38, *t*(90.9) = −9.91, *p* < .001) or for correct moves only (Exp. 1: beta = −0.17, *t*(1408.1) = −2.62, *p* = .009; Exp. 2: beta = −.11, *t*(3937.8) = −2.62, *p* = .009).

We further explored how the degree of schematic eye movements at retrieval (*w*_*moveProb*_) was related to participants’ responses. One possibility is that schematic eye movements at retrieval simply indicate that participants are preparing to make a schema-consistent guess on that trial. If that is the case, we should see that participants select the most probable moves at recall when *w*_*moveProb*_ is high. Alternatively, schematic eye movements at retrieval could indicate that participants were using their schema to generate potential moves in hopes of being able to recognize the correct move from episodic memory, in which case participants will be able to report the correct move even if it was not the most probable move for this board position. We tested this prediction by measuring the relationship between schematic eye movements at retrieval and the probability of the **selected** move, separately for correct and incorrect trials. We conducted a linear mixed effect regression, predicting probability of selected move from *w*_*moveProb*_, with a random subject intercept. When a move was incorrectly recalled, schematic eye movements strongly predicted the probability of the selected move (Exp. 1: beta = 0.58, *t*(1487.3) = 22.79, *p* < .001, Exp. 2: beta = 0.28, *t*(3246.3) = 21.16, *p* < .001), suggesting that on these trials schematic eye movements were used to guess a likely move in the absence of episodic memory. When a move was correctly recalled, however, schematic eye movements were not related to the probability of the selected move (Exp. 1: beta = .05, *t*(1407.9) = 1.40, *p* = 0.162, Exp. 2: beta = −.003, *t*(3934.0) = −0.17, *p* = .865). This provides evidence that schemas can be used strategically to retrieve episodic memories, rather than simply biasing guessing toward probable outcomes.

### Prediction Accuracy, But Not Probability, Reduces Schema Use at Retrieval

We next examined whether participants’ retrieval strategy (as measured through eye movements) differed for probable versus improbable moves and for predicted versus unpredicted moves. We conducted a linear mixed-effects regression with the schematic eye movement at retrieval (*w*_*moveProb*_) as the outcome variable and prediction accuracy (from eye movement before the move shows up), the probability of the move (according to the gameplay model), and their interaction as predictors, with a random subject intercept. In Exp. 1, both move probability and prediction accuracy significantly impacted schematic eye movement, but in the opposite directions (Figure 4b). Higher move probability was associated with more schematic eye movement (beta = 0.05, *t*(2890.0) = 3.30, *p* = .001), whereas low prediction accuracy was associated with more schematic eye movement (beta = −1.94, *t*(2897.2) = −9.75, *p* < .001). The interaction between move probability and prediction accuracy was not significant (beta = 0.01, *t*(2893.3) = 0.657, *p* = .511). In Exp. 2, move probability had no main effect on schematic eye movement (beta = −.003, *t*(7222.9) = −0.02, *p* = .811), while low prediction accuracy was associated with more schematic eye movement (beta = −0.18, *t*(7237.4) = −15.40, *p* < .001). There was a significant interaction between the two measures (beta = 0.04, *t*(7250.0) = 2.99, *p* = .003), such that more probable moves led to more schematic eye movement only if the prediction was accurate. Note that the direction of these effects reveals an interesting dissociation between prediction and probability: the moves which relied least on schematic retrieval were those that were simultaneously predicted and also low probability, a combination that would be difficult to observe without our independent manipulation of these two factors. For moves with large prediction errors, the effect of move probability was inconsistent across experiments, with *w*_*moveProb*_ increasing for higher probability moves only in Exp. 1.

Since we found that prediction accuracy decreased *w*_*moveProb*_ and that lower *w*_*moveProb*_ predicted more accurate responses, we tested whether reduced schematic eye movements mediated the memory benefits of accurate prediction. In both experiments, this mediation was significant (Exp. 1: bootstrapped indirect effect = 0.023, 95% CI = [0.176, 0.03], *p* < .001; Exp. 2: bootstrapped indirect effect = 0.0341, 95% CI = [0.028, 0.04], *p* < .001). This suggests that making accurate predictions during encoding is associated with the creation of precise episodic memories, allowing participants to rely less on schema at retrieval. On the other hand, move probability did not significantly reduce schematic eye movements in either Exp. (and in fact increased them in Exp. 1), providing evidence that better memory for probable moves does not rely on this same mechanism of facilitated episodic encoding.

One potential explanation of our prediction-related memory effect is that, when a move is shown at a participant’s gaze position, it increases the total encoding time for the move and therefore improves memory simply through additional exposure. This would be consistent with our finding that accurate predictions resulted in strong episodic memory. If memory is solely driven by the total encoding time, we would expect that the time spent looking at the move after it was shown should predict memory performance, potentially even more strongly than prediction accuracy. We conducted a mixed effect logistic regression predicting memory from fixation on the correct move during the encoding period after the move appeared, with a subject random intercept. We found that longer fixations on the move were not significantly associated with better memory in either experiment (*p* > .427). We additionally performed a Bayesian analysis, comparing the logistic regression model with fixation duration (and a per-subject random intercept) to a null model with only per-subject random intercepts. The Bayes factor for the fixation-duration model was 0.181 in Exp. 1 and 0.055 in Exp. 2, providing moderate to strong evidence in favor of the null model. This suggests that there is something special about pre-stimulus fixations to the correct move location in the prediction phase that drive subsequent memory.

## DISCUSSION

When experiencing events that unfold over time in naturalistic settings (Chen et al., 2017; Lee & Chen, 2021), our episodic memories are scaffolded by our knowledge of the world that we have built through repeated experiences (Baldassano et al., 2018; Masís-Obando et al., 2022). One core benefit of having a schema for an event is that it enables us to make predictions, which can have important implications for memory (Antony et al., 2021; Rouhani et al., 2018). This study aimed to measure the specific impact of successful and unsuccessful predictions on subsequent memory by deconfounding prediction accuracy from stimulus probability. We accomplished this using a novel paradigm in which we measured predictions using real-time eye-tracking and generated stimuli that were consistent or inconsistent with these predictions. Overall, the results support a model in which prediction accuracy and stimulus probability contribute to better memory through different mechanisms; accurate prediction facilitates the formation of a precise episodic memory that is recalled directly, while probable stimuli were more likely to be reconstructed through a schema-based process at retrieval.

### Accurate Predictions Facilitate Memory

We found that confirming a participant’s prediction improved later memory, whether or not the predicted stimulus was actually probable according to the gameplay model. Decades of research studies have examined how schemas help memory (Anderson, 1981). For schema-consistent information, like a pan on a stove, schemas not only facilitate rapid consolidation during encoding (Sommer et al., 2022), but also allow people to come up with guesses at retrieval that could serve as cues for recognition (Anderson & Pichert, 1978; Watkins & Gardiner, 1979). Recent work has argued for a central role of prediction in memory, showing that merely making a schema-consistent prediction is associated with better memory (Huang et al., 2023), and that how schema-inconsistent information is remembered depends on the strength of expectation and prediction error (Quent et al., 2021). Our results show that schematic knowledge can in fact improve memory through two separable mechanisms: by enhancing precise memory encoding through more accurate predictions, and by steering retrieval processes toward likely outcomes.

We found no evidence for improved memory from large prediction errors as reported in previous studies (Antony et al., 2023; Bein et al., 2021; Greve et al., 2017; Rouhani et al., 2018). One potential explanation for the lack of effect is the element of surprise in the study, where participants’ predictions were violated about 50% of the time. It is possible that in previous studies prediction errors benefit memory because they are rarer and more surprising. However, in a recent study which found a significant benefit of incongruency on memory (Quent et al., 2022), the amount of congruent and incongruent trials was balanced, and the lack of prediction error effect is unlikely solely due to the prediction errors being more surprising in previous studies. It is important to note, however, that many studies showing benefits of prediction error use reward paradigms (Jang et al., 2019; Rouhani et al., 2018), in which prediction errors provide a critical learning signal for improving mental models or action policies. Prediction error in the current study does not provide any new information about rewards or the rules of the game, and therefore may not trigger processes that enhanced memory in these studies. Additionally, outside the context of reinforcement learning, the effects of prediction error on memory have been less clear. In research on schema, participants are often shown object in a congruent or incongruent context (Quent et al., 2022; van Kesteren et al., 2013), where incongruent pairs are thought to generate prediction errors. Although some studies have shown better memory for schema-incongruent pairs (Quent et al., 2022), many have found the opposite result (Höltje & Mecklinger, 2022; Ortiz-Tudela et al., 2021; Poskanzer et al., 2025; van Kesteren et al., 2013). In addition, it is worth noting that research on both reinforcement learning and schema tend to use recognition or forced-choice memory tests (Greve et al., 2017, 2019), and research has shown better memory for unexpected items during recognition, but not recall (Lew & Howe, 2017). This paper therefore supports the view that the impact of prediction error on memory is more nuanced than previously assumed (Bein et al., 2023), and further research is needed to find the contexts in which prediction error benefits memory. One factor that could impact memory effects is the level of cortical hierarchy in the brain in which prediction errors occur. Previous studies have demonstrated that information accumulates at increasing timescales as it moves from sensory cortex into higher-level regions like prefrontal cortex (Hasson et al., 2015), and that there is a corresponding increase in the timescale of predictions (Lee et al., 2021; Tarder-Stoll et al., 2023). The current study relies on predictions that likely rely on higher-order regions such as medial prefrontal cortex (Hasson et al., 2015), and the effect of prediction error on memory might be different for lower-level predictions such as perceptual oddballs (Strange & Dolan, 2001). An alternative account for the observed benefit of prediction accuracy on memory is that this arises purely by increasing the effective amount of encoding time for the move stimulus, which is well known to improve memory in general (Murdock, 1974). It is possible that part of the mechanism through which prediction improves memory is by effectively allowing additional “pre-stimulus encoding” of the move and relevant visual features if the stimulus can be accurately anticipated before it appears. Prediction can also allow a participant to spend more time fixating on the move once it appears, which could allow for more extensive encoding (through processes not related to prediction per se). However, we found that this additional fixation time on the stimulus itself was not the route by which prediction impacted memory in our study; the amount of time spent fixating on the move after it appeared was not related to subsequent memory. This suggests that, if additional encoding time is playing a role, it is only the early pre-activation of stimulus content generated by predictive processes. We therefore argue that a generic encoding-time explanation fails to account for the specific impact of pre-stimulus anticipation that we observed in our study.

### Accurate Predictions Led to Reduced Reliance on Schema at Retrieval

Past literature has shown that eye-movements during retrieval represent meaningful temporal contexts of the memory representation (Kragel & Voss, 2021). Using eye movements at retrieval to detect when participants were using a schema-based reconstruction strategy, we found that this strategy was used more often for moves that were poorly predicted during encoding. We interpret the findings as evidence that that prediction confirmation leads to an enhanced episodic encoding process (Ramey et al., 2022), perhaps through facilitated processing of the expected stimulus (Sommer et al., 2022), such that they were recalled without having to search through schema-consistent possible moves. In addition, the positive emotional response evoked by correct predictions might make the stimulus more salient (Lee & Sternthal, 1999). Another potential mechanism could be related to how predicted and probable information were consolidated (van Kesteren et al., 2013). Future research could further investigate how memory encoding process and the resulting memory representation differs for predicted and unpredicted items, to better understand how accurate predictions facilitate precise episodic memory.

The finding that accurate prediction promotes forming a robust episodic memory is particularly relevant to theories of how people remember schema-consistent and schema-inconsistent information. For example, the “Schema-Linked Interactions between Medial prefrontal and Medial temporal lobe” model (van Kesteren et al., 2012) proposes that schema-consistent information will be remembered through reactivation of the schema whereas schema-inconsistent information will be remembered through retrieval of a specific instance memory. Our results are partially consistent with this model, in this sense that moves with high probability (consistent with the game schema) relied more on a generate-and-recognize strategy during recall (Watkins & Gardiner, 1979), though this effect was found only for accurately predicted moves in Exp. 2. This potentially saves attentional resources and allows them to focus on episodically encoding improbable moves (which cannot be easily accessed via a schema-based strategy at retrieval). This suggests that participants might create strong episodic memories only for improbable moves that they know will be difficult to generate at retrieval. This strategy may be especially useful when the task is more difficult, explaining why it is used more in Exp. 1 (which requires memorization of many more low-probability moves). Our findings are also consistent with prior work showing efficient encoding of schema-consistent information, but at the cost of memory precision (Bellana et al., 2021).

However, we also showed that accurate predictions (which are more likely to occur for schema-consistent moves) created strong episodic memories that could be retrieved without engaging schematic processes. Our findings could potentially explain why Quent et al. (2022) found that both schema-consistent and inconsistent items were associated with better recollection, if participants were able to make accurate predictions for the schema-consistent items. Future studies should consider the impact of prediction confirmation when designing experiments, since parameters such as the interval between context and item might facilitate or inhibit predictions and impact schema effects on memory.

### Methodological Implications

Finally, this work developed two methodological advances that can provide new insights in memory research and related fields. Although gaze-contingent paradigms have been used to study vision (Rayner, 1975) and social cognition (Wang et al., 2020; Wilms et al., 2010), and eye-tracking methods have been used in memory research (Clewett et al., 2020; Ramey et al., 2020; Wynn et al., 2019, 2020), adaptive paradigms with real-time eye-tracking have not previously been applied to memory research. Our work demonstrates that it is possible to track predictions over a large space of potential actions (on a 4 × 9 game board) to control when prediction errors occur, isolating the impact of prediction errors from factors such as outcome likelihood or reward magnitude. A common challenge in studying the impact of schematic knowledge is that it is difficult to disentangle encoding-time and retrieval-time mechanisms, motivating manipulations such as changing the schema between encoding and retrieval (Anderson et al., 1983; Bransford & Johnson, 1972). Our study established a novel method of disentangling these two kinds of processes, and also showed that eye movements during retrieval can index not only episodic memory of the item (Wynn et al., 2019), but also the degree to which schematic knowledge is being used to search for a memory. Both of these methods can be applied to fields including attention, learning and decision making. For example, real-time eye-tracking could be used to dissociate reward and prediction error in learning research (Rouhani et al., 2018), and eye movements during decision making could potentially reveal what strategies (e.g., episodic memory, model-free learning, model-based learning) were used, in addition to modeling the behavioral choice people made (Nicholas et al., 2022). Similarly, in studies of visual search (Castelhano & Heaven, 2011; Wynn et al., 2020), our modeling approach could provide insights into how different sources of information might be used.

To conclude, the current study used a complex board game in combination with real-time eye-tracking to test how prediction accuracy and stimulus probability separately contribute to memory. We found that both prediction accuracy and stimulus probability lead to better memory, but through different mechanisms: prediction accuracy boosts the formation of episodic memory, whereas stimulus probability benefits memory by through schema-based inference at retrieval. This study is part of a recent movement to use games to study cognition (Allen et al., 2023), since they probe more complex processes than traditional designs while still allowing for precise quantitative modeling. There are especially exciting possibilities for studying schematic prediction and memory with these paradigms, since both participants and computational models can make meaningful predictions about upcoming moves, even for novel board positions. Since moves correspond to spatial positions in 4-in-a-row, eye-tracking can provide new insight into predictive processes and adaptive experimental designs. Future work in this field should continue to explore the advantages of using game-based paradigms to study the perception and memory of naturalistic sequences.

## ACKNOWLEDGMENTS

We thank the editors and reviewers for providing constructive feedback for improv. ing the paper. We also thank past and current members of the Alyssano lab for feedback and discussion.

## FUNDING INFORMATION

The project is funded by National Institute of Health #R01EY034436.

## AUTHOR CONTRIBUTIONS

J. H. and C. B. designed research; J. H., E. F., Y. L., M. K., R. E., H. Z. performed research; J. H. and C. B. analyzed data; J. H. and C. B. wrote the paper.

## DATA AVAILABILITY STATEMENT

The data and code for the study can be found in https://osf.io/cqptj/.

## Supplementary Material


