# Supplementary material for: Accurate Predictions Facilitate Robust Memory Encoding Independently From Stimulus Probability
Source: Open Mind (Camb). 2025 Jul 26;9:940–58. doi: 10.1162/opmi.a.14 (PMC12373454; doi:10.1162/opmi.a.14)
Supplement: Supplementary file 1 [file opmi-09-940-s001.docx]

**Supplementary Information**

**Including prediction confidence to predict memory performance**

In our pre-registration, we planned to predict memory accuracy, reaction time, and confidence based on three regressors: in addition to the schema-consistency of the move (move probability) and prediction accuracy, which we reported in the paper, we also proposed to include another model-free measure of prediction: prediction confidence. Overall, we found that incorporating prediction confidence did not change the main findings of our study and we chose not to include this measure in the main text, but for completeness we report these analyses below.

Prediction confidence measured the extent to which a participant spent time looking at specific empty squares versus uniformly fixating across all empty squares pre-stimulus presentation. High values of prediction confidence indicate that a participant spent a large fraction of the trial looking at only a small number of empty squares, indicating a strong prediction about the upcoming move. As in Huang et al., (2023), we compute this as the expected information gain between a uniform distribution over all empty squares and the fixation distribution. Given the fixation time $T\left( x_{i} \right)$ for each square $x_{i}$, we define $P(empty)$as the fraction of the 10 or 8-second window during the initial board phase spent fixating on empty squares, and $P\left( x_{i} \right)=T\left( x_{i} \right)/P(empty)$ as the normalized fixation distribution over empty squares. The information gain from a fixation is 0 for fixations on occupied squares, and for fixations on empty squares reflects the entropy difference between a uniform distribution and the fixation distribution. Therefore, we define:

Prediction confidence $=P(\mathrm{empty}) \cdot\left( \log\left( N_{\mathrm{empty}} \right)-\sum_{i}^{N} P\left( x_{i} \right)\log P\left( x_{i} \right) \right)$

Refer to figure 6 of Huang et al., (2023) for visualization of some example boards with high and low prediction confidence.

We conducted linear mixed effect logistic regression model, predicting memory (accuracy, reaction time, and confidence) from move probability, prediction accuracy, prediction confidence, and the interaction between prediction accuracy and move probability, with subject random intercept.

In study 1 and 2, both move probability and prediction accuracy significantly lead to better memory (p < .001). In both studies, prediction confidence also led to better memory, although the effect is only marginally significant in study 2 (study 1: beta = 0.29, z = 2.87, p = .004; study 2: beta = 0.085, z = 1.71, p = .09). For the (memory) confidence measure, we found that move probability (t = 2.098, p = 0.04) and prediction accuracy (t = 2.767, p = 0.006), and prediction confidence (t = 2.51, p = 0.01) all led to more confident memory in study 1. Similarly, in study 2, all of the factors led to higher confidence (move probability: t = 8.05, p<.001, prediction accuracy: t = 3.71 p <. 001, prediction confidence: t = 3.33 p < .001). None of the interactions were significant. For reaction time, prediction confidence was strongly associated with faster reaction time in study 1 (t = -3.524, p < .001) while the other effects were in the same direction but not significant (move probability: t = -1.37, p = .171; prediction accuracy: t = -1.75, p = .079). In study 2, move probability, prediction accuracy, and prediction confidence all predicted faster reaction time: move probability (t = -3.96, p <.001), prediction accuracy (t = -2.01, p = .044), prediction confidence (t = -7.27, p < .001). Overall, these results showed that making confident predictions (spending a lot of time focusing on a few empty squares during encoding) lead to better memory, higher confidence, and faster reaction time, while the effects of accurate prediction and move probability remained the same as the main paper.

**Including prediction confidence to predict retrieval strategy**

In the pre-registration, we also mentioned that we would predict eye-movement measures at retrieval based on the schema-consistency of the move, prediction confidence, and prediction accuracy. As a result, we also incorporated prediction confidence into the regression predicting $w_{moveProb}$ (retrieval schematic eye movement) and $w_{correctMove}$. We conducted linear mixed effect regressions to predict $w_{moveProb}$ from move probability, prediction accuracy, and prediction confidence, and the interaction between prediction accuracy and move probability. In study 1, higher move probability led to higher $w_{moveProb}$ (t = 3.3, p = .001), and higher prediction accuracy led to lower $w_{moveProb}$ (t = -9.82, p < .001). Prediction confidence did not have a significant effect on $w_{moveProb}$ (t = 1.558, p = .119). The interaction between move probability and prediction accuracy was not significant. In study 2, move probability did not lead to a change in $w_{moveProb}$, but prediction accuracy decreased $w_{moveProb}$ (t = -15.57, p < .001), and prediction confidence increased $w_{moveProb}$ (t = 2.42, p = .016). There is a significant interaction between move probability and prediction accuracy (t = 2.94, p = .003).

**Analyses based on prediction confidence and accuracy at retrieval**

In our pre-registration, we planned to compute three statistics for retrieval eye movements, each corresponding to a measure of prediction in the previous study (prediction coefficient, prediction confidence, prediction accuracy). In the analysis reported in the main paper, $w_{moveProb}$ in retrieval is obtained in a similar way as prediction coefficient during the encoding phase, which we reported in detail. It can be considered as a model-based measure of how much schema is being used during retrieval.

We also proposed to look at applying the “prediction confidence” (described in the first section of the supplementary material) and “prediction accuracy” (described in the main paper) measures to retrieval, as model-free measure of retrieval strategy. A high “prediction accuracy” at retrieval would mean that a participant spent a large fraction of the time during recall looking at the correct move. A high “prediction confidence” at retrieval would mean that participants look at only a small number of empty squares during retrieval (low entropy), potentially indicating that they have the subjective experience of a precise episodic memory (whether or not that memory is accurate). To avoid confusion, here we refer to retrieval “prediction confidence” and “prediction accuracy” as “retrieval fixation confidence” and “retrieval fixation accuracy,” respectively.

retrieval fixation confidence $=\left( \log\left( N_{\mathrm{empty}} \right)-\sum_{i}^{N} P\left( x_{i} \right)\log P\left( x_{i} \right) \right)$

Note that, unlike the prediction confidence measure described in the first section of the supplementary materials, we do not weight the retrieval fixation confidence based on the time spent fixating on empty squares – retrieval is self-paced, and we would therefore expect the most confident responses to occur quickly (with relatively little time spent looking at empty squares). Retrieval fixation confidence can be considered a model-free measure of how much participants were debating between different response options, indicating how easy it was to recall the move.

Using mixed-effects linear regression, we predicted memory accuracy, reaction time, and memory confidence from retrieval fixation confidence and accuracy, with random subject intercepts. We found that moves with high retrieval fixation confidence were more likely to be accurately recalled (study 1: z = 9.92, p < .001; study 2: z = 14.32, p < .001), with higher memory confidence (study 1: t = 15.04, p < .001; study 2: t = 18.45, p < .001), and faster reaction times (study 1: t = -22.98, p < .001; study 2: t = -40.93, p <. 001). Similarly, moves with high retrieval fixation accuracy were better recalled, with higher memory confidence and faster reaction time (all p < .001).

We then tried to predict retrieval fixation confidence from move probability and prediction accuracy (during encoding). We found that in both studies, accurately predicted moves showed higher retrieval fixation confidence (study 1: t = 7.55, p < .001; study 2: t = 7.62, p < .001). More probable moves also showed higher retrieval fixation confidence (study 1: t = 3.23, p = .001; study 2: t = 3.54, p < .001). These results suggest that for probable and/or predicted moves participants considered only a small number of possible responses before making their response. Based on our results in the main text, however, we hypothesize that these effects arise for two different reasons: accurate predictions facilitate precise episodic memory (allowing for quick retrieval of the correct square), while, for probable moves, the move will be one of the first that participants generate (and then recognize) using their schema knowledge.

**Pupillometry analyses**

We also planned in our pre-registration to examine effects of the predictors (prediction accuracy, move probability, and prediction confidence) on pupil size. This was unfortunately difficult with our paradigm – the participants were constantly moving their eyes to look at different pieces with different luminance at different angles from the center of the screen, which had strong effects on measured pupil size. We first interpolated the size of the pupil during blinking periods using the package MNE (Gramfort, 2013), and then temporally downsampled the pupil timecourse by a factor of 10 (to 100 datapoints per second). For all moves, we built a model to predict pupil size from the distance of the eye from the center of the screen, and the color of the move closest to the location of the eye. We then took the residual of the model and visualized the change as a function of time (Supplementary Figure S1). As can be seen from the figure, the confidence intervals were largely overlapping for moves with varying probability and prediction accuracy. We have therefore chosen not to proceed with additional pupillometry analyses for this paradigm.


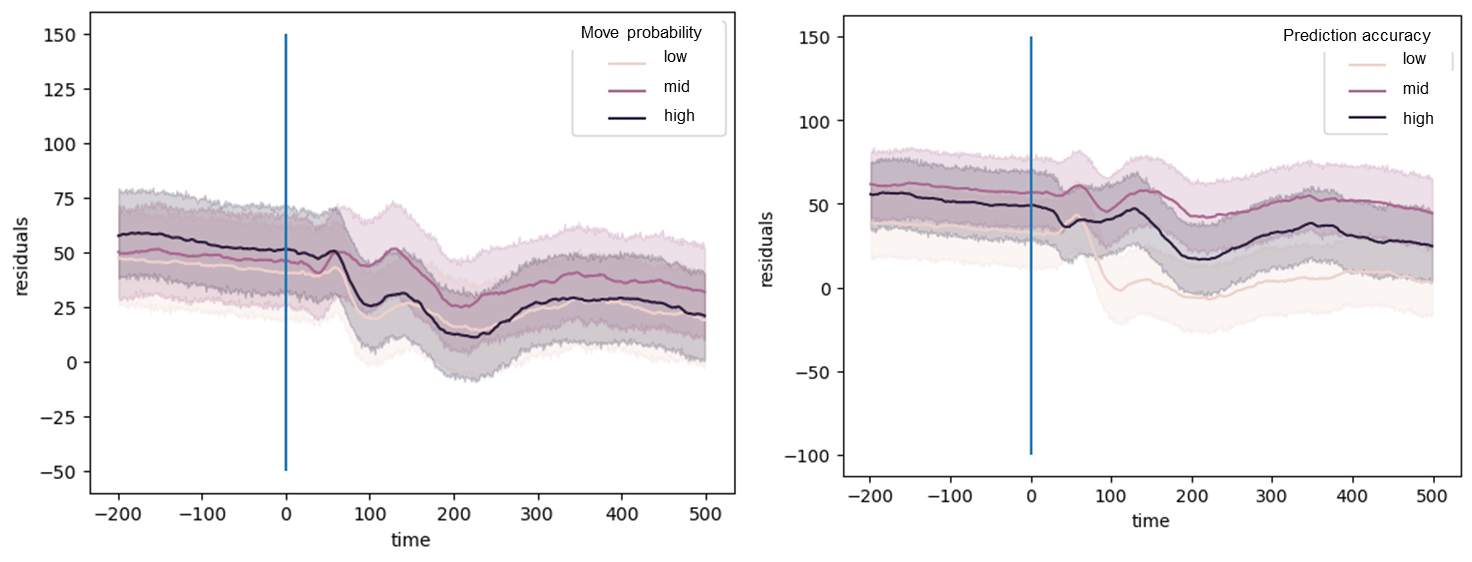


Supplementary Figure S1. Residual of pupil size as a function of time (1/100 sec). Different colors represent the tertiles of move probability and prediction accuracy. Blue marks represent the presentation of the stimulus. Error bars represent 95% confidence intervals.
